# A novel three-dimensional heterotypic spheroid model for the assessment of the activity of cancer immunotherapy agents

**DOI:** 10.1007/s00262-016-1927-1

**Published:** 2016-11-17

**Authors:** Sylvia Herter, Laura Morra, Ramona Schlenker, Jitka Sulcova, Linda Fahrni, Inja Waldhauer, Steffi Lehmann, Timo Reisländer, Irina Agarkova, Jens M. Kelm, Christian Klein, Pablo Umana, Marina Bacac

**Affiliations:** 1 Roche Pharmaceutical Research and Early Development, Oncology DTA, Roche Innovation Center Zurich, Wagistrasse 18, 8952 Schlieren, Switzerland; 2 InSphero AG, Wagistrasse 27, 8952 Schlieren, Switzerland; 3 Institute of Surgical Pathology, University Hospital Zurich, Schmelzbergstrasse 12, 8091 Zurich, Switzerland; 4 Institute for Biomedical Engineering, ETH Zurich, Wolfgang-Pauli-Strasse 10, 8093 Zurich, Switzerland; 5 Department of Biosystems Science and Engineering, ETH Zurich, Mattenstrasse 26, 4058 Basel, Switzerland

**Keywords:** Cancer immunotherapy, 3D model, Heterotypic spheroid model, T cell bispecific antibodies, Interleukin-2 immunocytokine fusion, Tumor–stroma interaction

## Abstract

The complexity of the tumor microenvironment is difficult to mimic in vitro, particularly regarding tumor–host interactions. To enable better assessment of cancer immunotherapy agents in vitro, we developed a three-dimensional (3D) heterotypic spheroid model composed of tumor cells, fibroblasts, and immune cells. Drug targeting, efficient stimulation of immune cell infiltration, and specific elimination of tumor or fibroblast spheroid areas were demonstrated following treatment with a novel immunocytokine (interleukin-2 variant; IgG-IL2v) and tumor- or fibroblast-targeted T cell bispecific antibody (TCB). Following treatment with IgG-IL2v, activation of T cells, NK cells, and NKT cells was demonstrated by increased expression of the activation marker CD69 and enhanced cytokine secretion. The combination of TCBs with IgG-IL2v molecules was more effective than monotherapy, as shown by enhanced effects on immune cell infiltration; activation; increased cytokine secretion; and faster, more efficient elimination of targeted cells. This study demonstrates that the 3D heterotypic spheroid model provides a novel and versatile tool for in vitro evaluation of cancer immunotherapy agents and allows for assessment of additional aspects of the activity of cancer immunotherapy agents, including analysis of immune cell infiltration and drug targeting.

**Electronic supplementary material:**

The online version of this article (doi:10.1007/s00262-016-1927-1) contains supplementary material, which is available to authorized users.

## Introduction

Traditionally, anticancer treatments have been tested in classical two-dimensional (2D) culture systems, but these models suboptimally reproduce the complex morphological and histopathological features characteristic of tumor microenvironments, particularly concerning tumor–host and tumor–immune cell interactions, which limits their value as models for cancer immunotherapy drug development [1]. Currently, there is limited availability of in vitro models that adequately mimic in vivo conditions. Compared to 2D cultures, three-dimensional (3D) models better predict in vivo immune cell behavior [2], thereby providing a more robust system to evaluate immunotherapeutic agents.

Cells cultured in 3D culture systems (spheroids) are phenotypically different to those grown in 2D culture systems (monolayers), as shown by differential gene expression [3–10]. In particular, expression of genes encoding signal transduction proteins, and cell surface markers, and localization and oligomerization of these markers in 3D cancer spheroids were shown to be more similar to the in vivo environment [11, 12]. The 3D microenvironment, including the extracellular matrix (ECM), is profoundly altered in tumors [13, 14], and 3D spheroid models simulate these alterations [15]. In 3D spheroids, the development of extracellular matrix (ECM), the distribution of oxygen and metabolites, and cell proliferation gradients are more similar to those of cancer tissues than those of 2D culture models and more closely resemble avascular or intervascular tumor microregions [10, 16–20]. Therefore, 3D spheroid systems constitute a more physiological model for in vitro testing of drugs [12, 21–23]. In addition to structural features, 3D cultures offer temporal advantages because they also allow kinetic analysis of therapeutic activity, which is suboptimally assessed in 2D cultures owing to fast and efficient immune cell activation and target cell lysis (unpublished data).

The behavior of the immune cells is also affected by the infiltration dynamics within a 3D scaffold due to physical constraints and chemical cues. Infiltration of immune cells was studied in benign and malignant cell spheroids, where it was found that stimulation of human peripheral blood mononuclear cells (PBMCs) by tumor cells or IL-2 was necessary for the infiltration of PBMCs into the spheroids [24]. In 3D melanoma spheroids, tumor cells had a higher capacity to inhibit both T cell activation and proliferation than in 2D cultures, suggesting that spheroids support a more physiological immune-modulatory function than 2D cultures [25]. Cell-killing assays in 3D models have shown comparable results to in vivo experiments [26]. Tumor 3D homotypic spheroids were also shown to be a more appropriate model than classical 2D cultures for studying the cytotoxicity and infiltration of NK cells, allowing stable, reproducible, and long-term co-culture with immune cells and enabling the characterization of immune cell subpopulations [27].

We explored the versatility of a newly developed 3D heterotypic spheroid model composed of tumor cells, fibroblasts, and immune cells by studying drug targeting and immune cell infiltration, activation, and cytotoxicity in response to novel cancer immunotherapy agents currently in Phase I clinical trials (IgG-IL2v and T cell bispecific antibodies [TCBs]) as monotherapy and in combination.

IgG-IL2v is a monomeric IL2 variant fused to an IgG1 antibody with FcγR-silent Fc that is either targeted to carcinoembryonic antigen (CEA)–expressing tumors (CEA-IL2v), fibroblast activation protein-α-expressing fibroblasts (FAP-IL2v), or untargeted (DP47-IL2v = IgG-IL2v). The IL2v mutations abolish binding to the alpha subunit of the IL2 receptor (IL2Rα, CD25) and result in an IL2 variant (IL2v) that lacks the preferential activation of regulatory T cells (T_regs_) by normal IL-2, binding only to the IL2 receptor βγ complex and thus leads to activation and expansion of CD8+ effector, CD4+ helper T cells and NK cells in vitro and in vivo [28, 29]. Phase I clinical trials with CEA-IL2v as well as FAP-IL2v are currently ongoing (NCT02350673 and NCT02627274) [30, 31].

CEA-targeted TCB (CEA TCB) is a novel tumor-targeted T cell bispecific antibody that binds simultaneously to CEA overexpressed on many solid tumors (colorectal, pancreatic, gastric, lung, breast, and ovarian carcinomas) and to CD3 (epsilon chain, CD3e) present on T cells. In the presence of CEA-expressing target cells and T cells, treatment with CEA TCB leads to rapid formation of immunological synapses and T cell activation followed by T cell-mediated killing of tumor cells, T cell proliferation and cytokine release [32–34]. Phase I clinical trials with CEA TCB are currently ongoing (NCT02324257) [35]. FAP TCB is a fibroblast-targeted TCB that binds to FAP and the CD3e antigen.

## Materials and methods

### Cell lines and 3D heterotypic spheroid cultures

Human colon adenocarcinoma cells LS174T (ECACC 87060401) and LoVo (ECACC 87060101) were cultured in Eagle’s Minimum Essential Media (EMEM; Gibco^®^, Life Technologies, Zug, Switzerland) and DMEM (Gibco^®^, Life Technologies, Zug, Switzerland), respectively; MRC-5 normal fetal lung fibroblast cells (ATCC CCL-171) and CCD-18Co a normal colon fibroblast cell line (ATCC CRL-1459) were cultured in EMEM or fibroblast growth medium-2 (FGM™-2; Lonza, Basel, Switzerland). Media were supplemented with 10% FBS (Gibco^®^, Life Technologies, Zug, Switzerland), 2 mM GlutaMax (Gibco^®^, Life Technologies, Zug, Switzerland), 1% nonessential amino acid solution (NEAA, 100×, Sigma-Aldrich Chemie GmbH, Buchs, Switzerland), and 1 mM sodium pyruvate (Sigma-Aldrich Chemie GmbH, Buchs, Switzerland). Cells were cultured in a humidified atmosphere of 5% CO_2_ at 37 °C and split every 2–3 days at a ratio of 1:5 (LS174T and LoVo) and 1:2 (MRC-5, CCD-18Co).

3D heterotypic spheroid cultures were generated by hanging drop method using the GravityPLUS™ kit (InSphero AG, Schlieren, Switzerland) at a ratio of 1:50 (tumor cells to fibroblasts). Spheroids were cultured in FGM™-2 supplemented with 10% FBS. Cultures were incubated in a humidified atmosphere of 5% CO_2_ at 37 °C for 3 days. Spheroid compaction was confirmed by observation under a microscope, before transferring spheroids to GravityTRAP™ assay plates (InSphero AG, Schlieren, Switzerland).

PBMCs were isolated from healthy donors by standard Histopaque density gradient centrifugation and added to the supernatant.

For IL2 (Proleukin [aldesleukin], Novartis) and IgG-IL2v antibody monotherapy assays, heterotypic spheroids were incubated with PBMCs and IgG-IL2v antibody (100 nM) for 24 h. For TCB monotherapy assays, heterotypic spheroids were incubated for 24, 48, and 72 h with PBMCs (PBMCs to fibroblasts 10:1) and TCBs (50 nM). For IgG-IL2v and TCBs combination therapy assays, heterotypic spheroids were pre-incubated for 16 h with untargeted (DP47) IgG-IL2v (10 nM) and PBMCs (PBMCs to fibroblasts 10:1). On the following day, the heterotypic spheroids and PBMCs were washed with fresh media and then cultured for 24, 48, and 72 h in medium containing TCBs (50 nM). Non-treatment control assays were performed in the presence and absence of PBMCs.

### Targeting of IgG-IL2v in the heterotypic spheroid model

Fluorescently labeled CEA-targeted, FAP-targeted, and untargeted IgG-IL2v cytokines were used to monitor targeting in 3D heterotypic spheroids. 3D heterotypic spheroids were incubated for 16 h in media containing 100 nM labeled IgG-IL2v. Media were replaced to remove unbound IgG-IL2v before rinsing the cells in Dulbecco’s phosphate-buffered saline (DPBS) and fixing in 4% paraformaldehyde for 1 h at room temperature. Cells were embedded in mounting medium containing DAPI (InSphero AG, Schlieren, Switzerland) and transferred onto glass slides prior to 2-photon confocal microscopy. Two-photon confocal microscopy provided high-resolution imaging of the inner spheroid core and detection of the bound fluorescently labeled IgG-IL2v. Tumor and fibroblast compartments were identified based on morphology.

### Immunohistochemistry and quantification

For IHC analysis, heterotypic spheroids were embedded in paraffin wax. Staining was performed on 3-µm thick serial sections of the heterotypic spheroids mounted onto poly-L-lysine-coated glass slides. The morphology of the sectioned heterotypic spheroids was evaluated after H&E staining. IHC was performed by SophistoLab^®^ (Muttenz, Switzerland) using the horseradish peroxidase Bond Polymer Refine Detection kit (Leica Biosystems AG, Muttenz, Switzerland) and analyzed using the BOND-MAX instrument (Leica Microsystems Newcastle Ltd, Newcastle, UK) as per manufacturer’s guidelines. Primary antibodies used for staining were: anti-human CD3 (clone SP7, NeoMarkers Inc., Fremont, US); anti-human CEA (produced in-house), anti-human CD45 (clone 2B11 & PD7/26; Cell Marque Lifescreen Ltd, Rocklin, US), anti-human CD14 (ab45870, Abcam Ltd, Cambridge, UK), and antihuman Seprase monoclonal antibody (Clone D8) (Vitatex Inc., Stony Brook, US).

CEA+ (tumor cells), CEA− (fibroblasts), and CD3+ (infiltrated T cells) areas were quantified by digital immunostained slides using ImageJ software (National Institutes of Health). CEA+ and CEA− areas were quantified by normalization to the total heterotypic spheroid area. The CD3+ areas within tumor and fibroblast compartments were quantified by normalization to the total tumor and fibroblast-rich areas, respectively. Tumor and fibroblast compartments were identified by morphology. Statistical analysis was performed by Student’s *t* test.

### Cytokine/chemokine release by cytometric bead array

Cytokine/chemokine secretion in the supernatant was measured by flow cytometry, using the Cytometric Bead Array (CBA, BD Biosciences, Franklin Lakes, NJ, USA), according to the manufacturer’s guidelines. Supernatants from individual heterotypic spheroids were collected and stored at −20 °C. Supernatants were subsequently thawed, and 1 well (50 µL) and 5 pooled wells (150 µL) were analyzed under each treatment condition for IgG-IL2v monotherapy and TCB monotherapy/combination therapy experiments, respectively. The following CBA kits (BD Biosciences, Franklin Lakes, NJ, USA) were used: CBA human IFNγ Flex Set, CBA human Granzyme B Flex Set, CBA human RANTES Flex Set (D4), CBA human MIP-1β Flex Set (E4), CBA human TNF Flex Set, CBA human IL-1β Flex Set (B4), and CBA human IL-6 Flex Set. Samples were measured using the BD FACS Canto II, and analyses were performed using the Diva Software (BD Biosciences, Franklin Lakes, NJ, USA). Assays were performed in triplicate.

### Flow cytometry

Tumor/fibroblast (ratio 1:50) heterotypic spheroids were incubated with 5 × 10^4^ PBMCs per well. Following treatment, the external tumor layer of the heterotypic spheroids was dissociated by 5 min incubation at room temperature in enzyme-free, phosphate-buffered saline-based cell dissociation buffer (Gibco^®^, Life Technologies, Zug, Switzerland). The remaining central core of fibroblasts with residual tumor cells was dissociated by 10–20 min incubation with 0.64 mg/mL Dispase II and 1 mg/mL Collagenase D (Roche Diagnostics, Mannheim, Germany). The single-cell suspension was washed with DPBS and resuspended in the DPBS containing antibody mixture for cell staining. For each condition, 32 heterotypic spheroids were pooled and tested in triplicate.

Flow cytometry was performed using anti-CEA labeled with Alexa 488 (produced in-house), PerCPCy5.5 anti-human CD45 (Biolegend, San Diego, CA, USA), Brilliant Violet 421 anti-human CD56 (Biolegend, San Diego, CA, USA), Brilliant Violet 605 anti-human CD69 (Biolegend, San Diego, CA, USA), Brilliant Violet 605 Mouse IgG1, κ (kappa) Isotype (Biolegend, San Diego, CA, USA), and LIVE/DEAD^®^ Fixable Aqua Dead Cell Stain Kit (Life Technologies, Zug, Switzerland). Samples were measured using the BD FACS Fortessa. Analyses were performed using the Diva Software (BD Biosciences, Franklin Lakes, NJ, USA). Assays were performed in triplicate.

### Statistics

The statistical analysis was performed using GraphPad PRISM software version 6. Error bars represent the standard deviation in all graphs. Two-tailed, unpaired parametric *t* tests were performed by setting the confidence intervals to 95% (definition of statistical significance: *p* < 0.05).

## Results

### Generation of the heterotypic spheroids

**Fig. 1:**
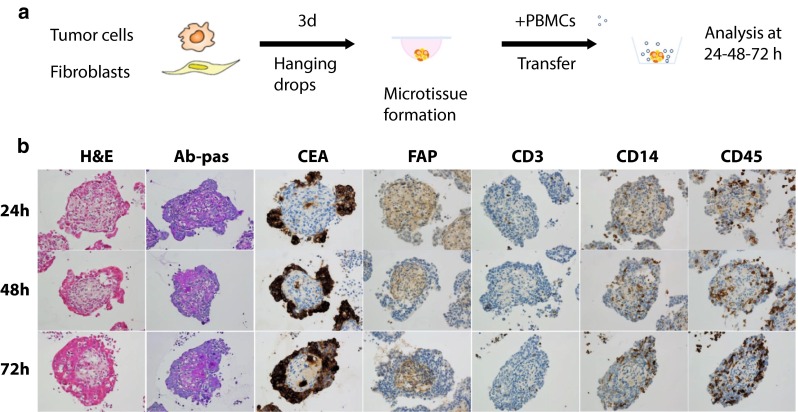
Heterotypic spheroids generation and histology **a** Heterotypic LS174T/MRC-5 spheroids were generated by co-culturing tumor cells (LS174T) and fibroblasts (MRC-5) by hanging drop method at a ratio of 1:50. Upon assembly and compaction, spheroids were transferred to a new assay plate and freshly isolated human PBMCs were added to the culture supernatant. **b** H&E, Ab-pas, and immunohistochemical (CEA, FAP, CD3, CD14, and CD45) staining at 24, 48, and 72 h on 3-µm thick serial sections of the LS174T/MRC-5 spheroids

An overview of the generation the heterotypic tumor/fibroblast/immune cell spheroids and the histology analysis is shown in Fig. 1a, b. During spheroid formation, tumor cells (LS174T) and fibroblasts (MRC-5) segregate in two different compartments. Tumor cells (identified by CEA+ staining) form an external peripheral layer, which surrounds the central core of fibroblasts (identified by FAP+ staining). The central core of fibroblasts secretes a mucopolysaccharidic extracellular matrix, as shown by the Ab-pas staining (Ab-pas+). The tissue microarchitecture also changes over time: Tumor cells, initially forming separate clusters, evolve into a continuous external layer that becomes thicker over time while the fibroblast compartment becomes more compact. This could potentially be due to contractile forces generated by fibroblasts and their lower proliferation rate as compared to the high proliferation rate of malignant tumor cells. When added to culture supernatants containing spheroids, the immune cells spontaneously infiltrate the heterotypic spheroids, as shown by CD45+ and CD14+ staining, which mark leukocytes and monocytes, respectively (Fig. 1b). The spontaneous infiltration of T cells (as detected by CD3+ staining) was generally found to be low (Fig. 1b).

Similar results were obtained with different cancer cell lines and different primary fibroblasts (Supplementary Figure 1), confirming that the novel heterotypic spheroids containing tumor, fibroblasts, and human immune cells provide a suitable and versatile model for the assessment of the effects of tumor- and fibroblast-targeted cancer immunotherapy agents.

### IgG-IL2v monotherapy-mediated targeting, infiltration, tumor cell lysis, and immune cell activation in the 3D heterotypic spheroid model

**Fig. 2:**
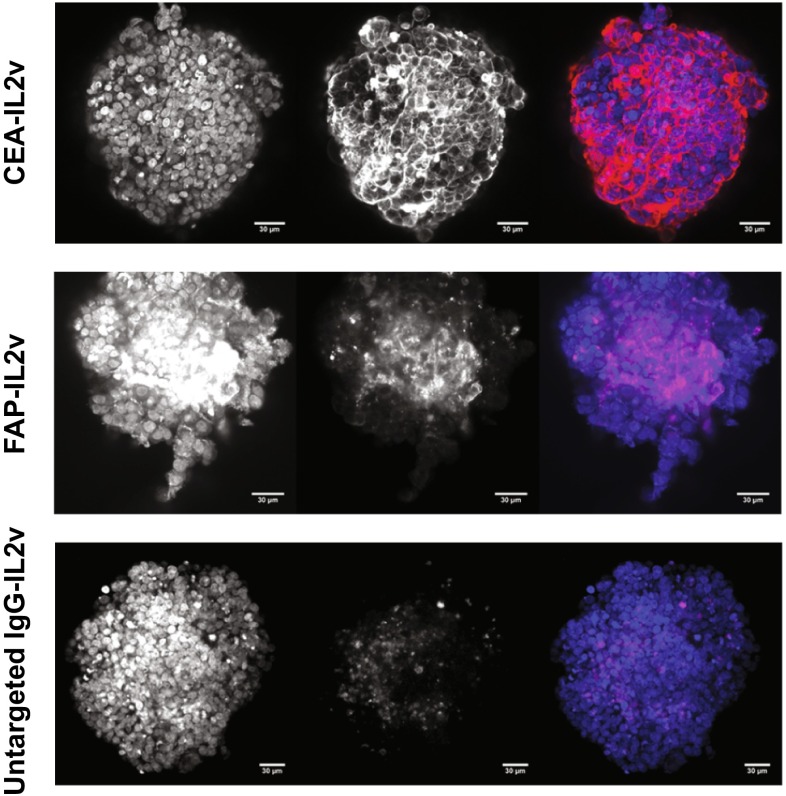
IgG-IL2v targeting. Heterotypic LoVo/MRC-5 spheroids incubated for 16 h with 100 nM of fluorescently labeled tumor-targeted IgG-IL2v (CEA-IL2v, colored *red*), fibroblast-targeted IgG-IL2v (FAP-IL2v, colored *red*), and the corresponding untargeted control (untargeted IgG-IL2v, colored *red*). Based on cell morphology, CEA-IL2v localized to the tumor cell compartment, FAP-IL2v localized to the fibroblast compartment, and the untargeted IgG-IL2v was not retained in any of the spheroid compartments as shown by the absence of localization with any of the cell types. *Blue* DAPI

Targeting of each of the IgG-IL2v molecules (CEA-IL2v, FAP-IL2v, and DP47-IL2v) to the various compartments of the 3D heterotypic spheroids was assessed using 100 nM of fluorescently labeled molecules (Fig. 2) CEA-IL2v penetrated the spheroid and remained targeted to the tumor cell compartment. FAP-IL2v penetrated the spheroid and remained targeted to the fibroblast-rich core, binding to fibroblast cells. Untargeted IgG-IL2v penetrated the spheroid but was not retained in any of the spheroid compartments. These results demonstrate the specificity of targeting of CEA- and FAP-targeted IgG-IL2v and show that the heterotypic spheroid model is suitable for the evaluation of tumor- and fibroblast-targeted immunotherapies.

**Fig. 3:**
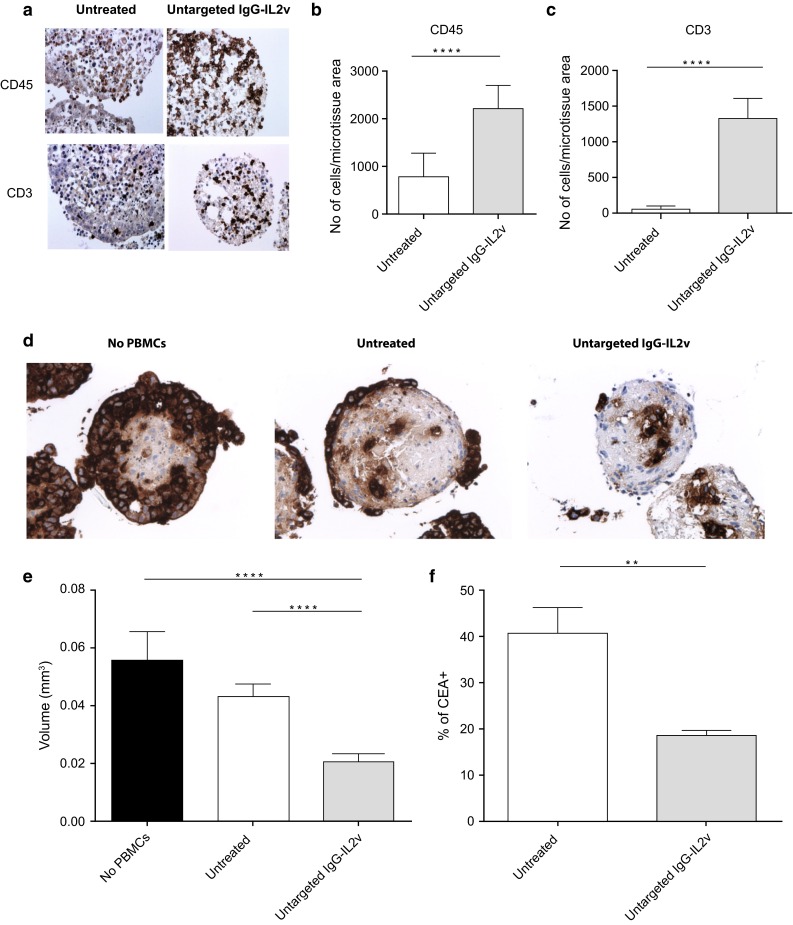
IgG-IL2v therapy-mediated effects. **a**–**c** Stimulation of immune cell infiltration after 24 h incubation of the heterotypic LoVo/MRC-5 spheroids with 100 nM untargeted IgG-IL2v. **a** Immunohistochemical (CD45, CD3) staining on 3-µm thick serial sections of the spheroids. The infiltration rates were calculated based on the number of CD45+ leukocytes (**b**) and CD3+ T cells (**c**) normalized to the spheroid area (two-tailed unpaired *t* test *p < 0.05, **p < 0.01, ***p < 0.001, ****p ≤ 0.0001, *N* = 3–10). **d**–**f** Tumor cell elimination in the heterotypic LoVo/MRC-5 spheroids after 24 h treatment with 100 nM untargeted IgG-IL2v assessed by CEA-staining 3-µm thick serial sections (**d**), spheroid size reduction (**e**) and flow cytometry CEA staining of dissociated spheroids (**f**) (two-tailed unpaired *t* test *p < 0.05, **p < 0.01, ***p < 0.001, ****p ≤ 0.0001, *N* = 3–10)

In order to study general effects mediated by untargeted IgG-IL2v on immune cell activation and infiltration in the heterotypic spheroid model, PBMCs and IgG-IL2v-antibodies (100 nM) were added simultaneously to culture supernatants containing the spheroids. Twenty-four-hour treatment with IgG-IL2v enhanced infiltration of immune cells, in particular T cells into the heterotypic spheroids, as detected by CD45 and CD3 staining, respectively (Fig. 3a–c). Comparison of untargeted IL-2v to tumor-targeted IL-2v and to Proleukin showed that all 3 compounds can induce similar amounts of leukocyte infiltration at equal exposure. Equal exposure to the different molecules was ensured by lack of washing steps after their addition (Supplementary Figures 2, 3).

In addition to assessing IgG-IL2v-mediated immune cell infiltration into the spheroids, we also evaluated tumor cell elimination in the spheroid mediated by IgG-IL2v-stimulated immune cells using three experimental readouts: histological examination, measurement of the reduction in spheroid size, and flow cytometry analysis (Fig. 3d–f). Compared with a control lacking PBMCs, 3D heterotypic spheroids incubated with PBMCs showed some spontaneous (therapy-independent) immune cell–mediated killing of tumor cells by 24 h, measured by CEA staining of tumor cells (Fig. 3d). Elimination of tumor cells was markedly enhanced by treatment with untargeted IgG-IL2v, which resulted in the complete elimination of the peripheral layer of tumor cells surrounding the inner fibroblast core. This treatment led to a significant reduction in spheroid size (2.1-fold, Fig. 3e) and a reduction in CEA+ cells detected by flow cytometry upon dissociation of heterotypic spheroids (by 1.8-fold, Fig. 3f).

**Fig. 4:**
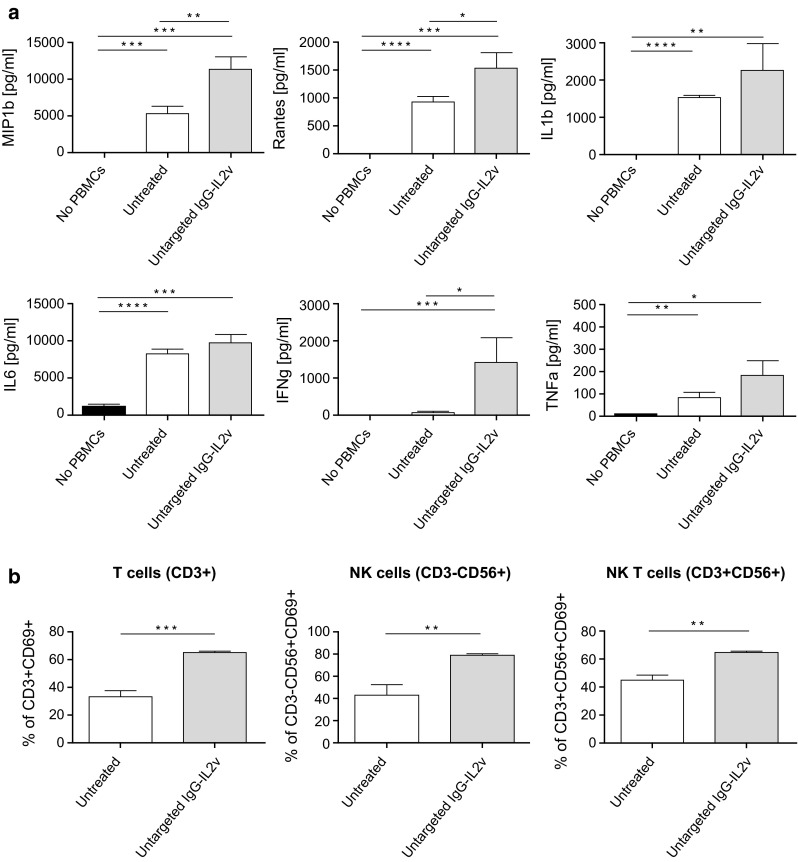
Cytokine release and activation of infiltrated immune cells in dissociated spheroids. Cytokine/chemokine secretion (CBA analysis, **a**) and immune cell activation (analysis CD69+ status) of subpopulations of infiltrated immune cells in dissociated spheroids (T-, NK-, and NKT cells, **b**) after 24-h treatment with 100 nM untargeted IgG-IL2v of heterotypic LoVo/MRC-5 spheroids (two-tailed unpaired *t* test *p < 0.05, **p < 0.01, ***p < 0.001, ****p ≤ 0.0001, *N* = 3)

In addition to tumor lysis, cytokine/chemokine secretion was also enhanced by the treatment with IgG-IL2v as compared with control cultures (Fig. 4a). Twenty-four-hour treatment of heterotypic spheroids with untargetd-IL2v led to increased cytokine/chemokine secretion, as shown by a significant increase in MIP-1ß, Rantes and IFNγ secretion and a more modest increase of IL1ß, IL-6, and TNFα. In line with the infiltration data, treatment of homo- or heterotypic spheroids with equal amounts of Proleukin, untargeted IgG-IL2v, or CEA-targeted-IL2v in the supernatants led to comparable cytokine/chemokine release (Supplementary Figure 4).

Upon dissociation of spheroids, flow cytometric analysis of the activation status of infiltrated immune cells showed an increase in the activation marker CD69 on T cells (CD3+), NK cells (CD3−, CD56+), and NK T cells (CD3+, CD56+) when treating with IgG-IL2v (Fig. 4b). In summary, IgG-IL2v treatment boosted the activity of different immune cell subsets and led to efficient elimination of tumor cells.

### Investigating the activity of TCBs in monotherapy and in combination with IgG-IL2v using the 3D heterotypic spheroid model

**Fig. 5:**
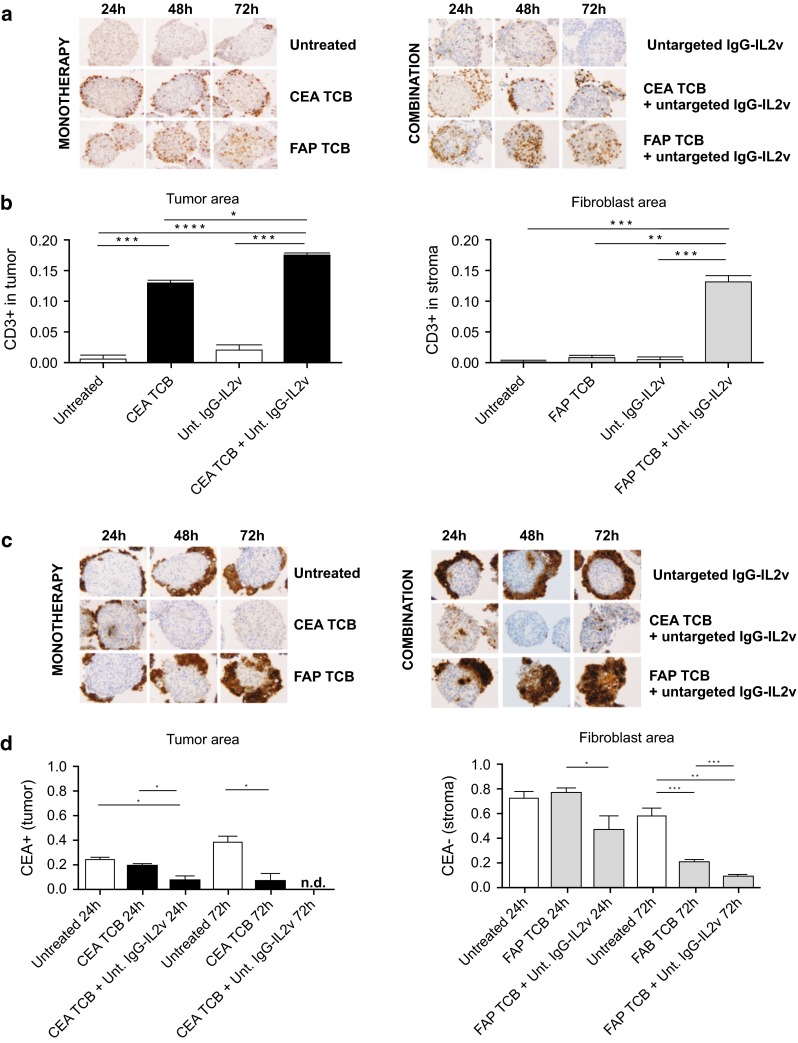
Immune cell infiltration and tumor cell elimination following treatment with CEA TCB and FAP TCB in monotherapy and in combination with IgG-IL2v. **a** CD3 staining of 3-µm thick serial sections of heterotypic LS174T/MRC-5 spheroids shows T cell infiltration and cross-linking to the tumor compartment (CEA TCB) and to the fibroblast compartment (FAP TCB) upon monotherapy with targeted TCBs (50 nM) and in combination with untargeted IgG-IL2v (10 nM) after 24, 48, and 72-h treatment. **b** T cell infiltration (CD3+ area) within the tumor area and fibroblast spheroid area was quantified at 24 h by normalization to the tumor and fibroblast-rich areas, respectively (two-tailed unpaired *t* test *p < 0.05, **p < 0.01, ***p < 0.001, ****p ≤ 0.0001, *N* = 3–4). **c** The CEA staining shows elimination of targeted spheroid areas upon monotherapy with targeted TCBs (50 nM) and in combination with untargeted IgG-IL2v (10 nM) after 24, 48, and 72 h in heterotypic LS174T/MRC-5 spheroids. **d** Reduction in the tumor and fibroblast areas in the heterotypic spheroids after treatment was quantified by CEA+ and CEA− staining, respectively (two-tailed unpaired *t* test *p < 0.05, **p < 0.01, ***p < 0.001, ****p ≤ 0.0001, *N* = 3–4)

Treatment of heterotypic spheroids co-cultured with immune cells added to supernatants with CEA TCB or FAP TCB resulted in target-specific cross-linking and retention of T cells to tumor cells or fibroblast areas (Fig. 5a, b), respectively. In particular, T cell cross-linking into the tumor area was significantly increased when spheroids were treated with CEA TCB as monotherapy (28.5-fold) and further increased when added in combination with IgG-IL2v (39.1-fold), as compared with the respective controls (Fig. 5a, b). Furthermore, CEA TCB-mediated cross-linking of T cells to tumor cells led to a selective elimination of CEA+ tumor areas 72 h post-treatment (Fig. 5c, d), and combination of CEA TCB IgG-IL2v further accelerated the elimination of tumor areas, reaching statistical significance as early as 24 h post-treatment (Fig. 5c, d).

Similarly, T cell cross-linking to fibroblasts was specifically increased when spheroids/immune cell co-cultures were treated with FAP TCB (numerical increase of 7.5-fold), with a significantly higher increase in combination with IgG-IL2v (140.1-fold), as compared with respective controls (Fig. 5a, b). The fibroblast area (CEA−) was reduced by FAP TCB treatment after 72 h (3.7-fold, Fig. 5c, d). As seen with CEA TCB, the combination with IgG-IL2v accelerated the killing process, resulting in a specific reduction in the fibroblast area that reached statistical significance at 24 h post-treatment and was further pronounced at 72 h (5.2-fold, Fig. 5c, d). These results indicate that the combination of IgG-IL2v with CEA TCB and FAP TCB enhances T cell infiltration and target-specific T cell cross-linking and retention into the spheroids as compared with TCB therapy alone, resulting in faster and more efficient elimination of tumor cells or fibroblasts bearing the target. The tumor-targeted TCB did not increase T cell cross-linking within the fibroblast compartment, and the fibroblast-targeted TCB did not increase T cell cross-linking within the tumor compartment, demonstrating target selectivity of the TCBs (Supplementary Figure 5).

**Fig. 6:**
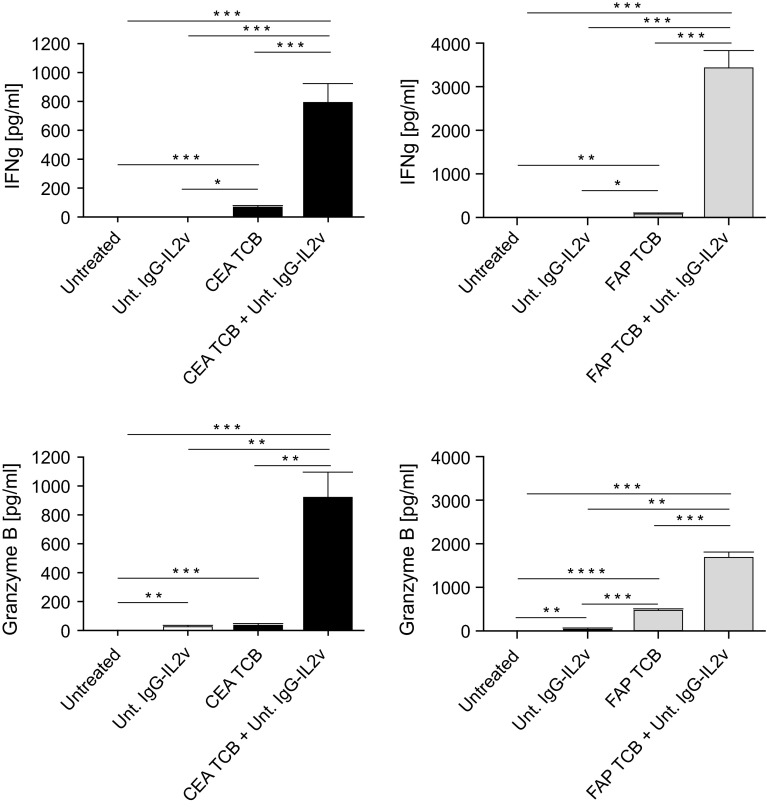
IFNγ and Granzyme B secretion following treatment with CEA TCB and FAP TCB in monotherapy and combination with IgG-IL2v. Cytokine release by heterotypic LS174T/MRC-5 spheroids cultured with PBMCs following treatment with CEA TCB or FAP TCB (50 nM) monotherapy and combination with untargeted IgG-IL2v (10 nM) after 24 h (two-tailed unpaired *t* test *p < 0.05, **p < 0.01, ***p < 0.001, ****p ≤ 0.0001, *N* = 3)

The heterotypic spheroid cultures also allow measurement of T cell effector function, by measurement of cytokines and chemokines in the culture supernatants. Release of the T cell effector molecules IFNγ and Granzyme B was significantly increased upon treatment with CEA TCB or FAP TCB as compared to untreated controls (Fig. 6), and IFNγ and Granzyme B release was significantly higher upon combination of TCBs with IgG-IL2v as compared to single agents, reflecting the stronger immune cell activation following combination treatment.

## Discussion

The 3D heterotypic spheroid model presented here provides a novel and versatile platform for testing of tumor- and fibroblast-targeted cancer immunotherapy agents.

The effects of a TCB antibody in a 3D homotypic spheroid model were recently reported by Hirschhaeuser et al. [36]. However, this homotypic 3D model lacked the simultaneous presence of tumor cells and fibroblasts. Therefore, despite providing valuable information, this homotypic model is suboptimal for the assessment of therapies targeted to either tumor or fibroblasts (stroma) and does not allow the analysis of immune cell infiltration and activity directed to one of the two compartments. Here, we report for the first time a novel 3D heterotypic spheroid model composed of tumor cells, fibroblasts, and immune cells. This allows for cell–cell and cell–matrix interactions in a 3D microenvironment where immune cells can infiltrate and migrate toward both tumor cells and fibroblasts, thereby improving the predictability of in vitro testing of immunotherapy agents. This novel 3D heterotypic spheroid model more closely resembles the complexity of tumors than either homotypic 3D spheroid models or 2D culture systems. Two-dimensional models are useful for large screening assays of candidate therapeutics to select potential drug candidates as killing of tumor target cells or fibroblasts in 2D is very efficient. However, the 3D model can offer a more realistic picture of the in vivo activity of a potential drug candidate.

The 3D spheroids presented in the current manuscript represent a novel heterotypic model; however, limitations include the use of tumor and fibroblast cell lines and PBMCs derived from healthy individuals added at high E:T ratios. Such 3D models could be further optimized by using patient-derived tumors containing TILs with physiological E:T ratios. Alternatively, infiltration and activation of TILs, rather than healthy PBMCS could be investigated, given that TILs are frequently suppressed and show suboptimal activity as compared to PBMCs derived from healthy donors.

Specific targeting and retention to tumor and fibroblast spheroid areas was demonstrated using tumor- and fibroblast-targeted IgG-IL2v (CEA-IL2v and FAP-IL2v, respectively), along with enhanced leukocyte infiltration, activation, and subsequent elimination of tumor cells. Treatment with tumor- or fibroblast-targeted TCB antibodies (CEA TCB and FAP TCB, respectively) led to efficient target-specific cross-linking and retention of T cells to tumor cells or fibroblasts, respectively. This resulted in effective elimination and reduction in the corresponding spheroid areas. The combination of both TCBs with IgG-IL2v was more efficacious than either monotherapy alone, as shown by an increase of immune cell infiltration and activation, faster elimination of target cells, and enhanced IFNγ and Granzyme B secretion.

Taken together, the 3D heterotypic model presented here enables a comprehensive in vitro evaluation of immune cell infiltration and target cell elimination after therapeutic intervention, which would not be possible using 2D systems. The study also demonstrates that tumor- and fibroblast-targeted IgG-IL2v and TCBs are promising candidates for combination treatments in cancer immunotherapy.

## Electronic supplementary material

Below is the link to the electronic supplementary material.

Supplementary material 1 (PDF 458 kb)

## Abbreviations

2D: Two-dimensional
3D: Three-dimensional
CBA: Cytometric bead array
CEA: Carcinoembryonic antigen
CEA TCB: Carcinoembryonic antigen-targeted T cell bispecific antibody
DPBS: Dulbecco’s phosphate-buffered saline
ECM: Extracellular matrix
EMEM: Eagle’s minimum essential medium
FAP: Fibroblast activation protein-α
FGM-2: Fibroblast growth medium-2
TCB: T cell bispecific antibody
T_regs_: Regulatory T cells
