# Supplementary material for: A novel three-dimensional heterotypic spheroid model for the assessment of the activity of cancer immunotherapy agents
Source: Cancer Immunol Immunother. 2016 Nov 17;66(1):129–40. doi: 10.1007/s00262-016-1927-1 (PMC5222939; doi:10.1007/s00262-016-1927-1)
Supplement: Supplementary file 1 — Supplementary material 1 (PDF 458 kb) [file 262_2016_1927_MOESM1_ESM.pdf]

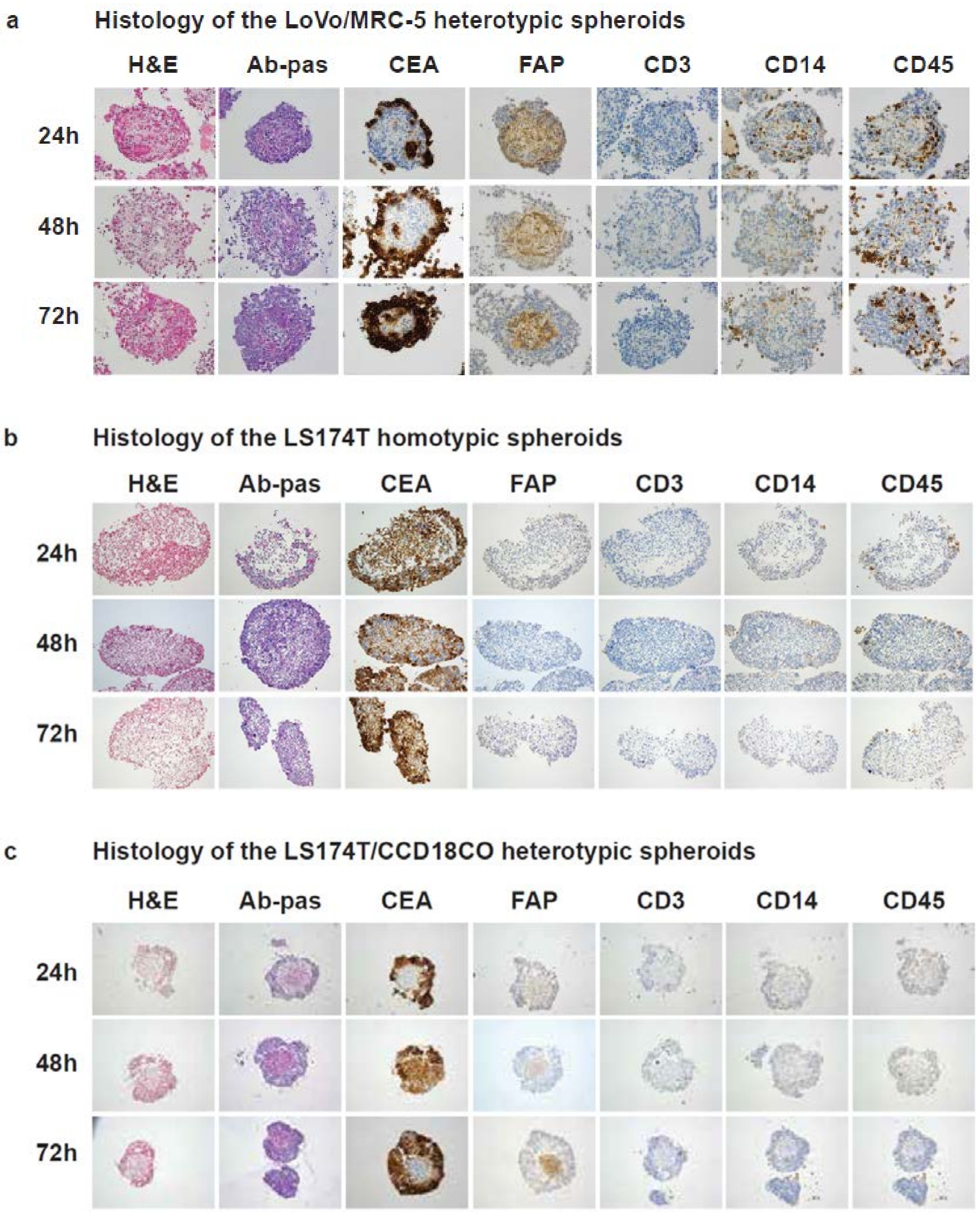

Supplementary Figure 1 Histology of homo- and heterotypic spheroids. Hematoxylin-eosin, ab-pas, and immunohistochemical (CEA, FAP, CD3, CD14 and CD45) staining were performed at 24 h, 48 h, and 72 h on 3-µm thick serial sections of LoVo/MRC-5 heterotypic spheroids (**a**), LS174T homotypic spheroids (**b**) and LS174T/CCD-18Co heterotypic spheroids (**c**) by hanging drop method at a ratio of 1:50. Similar to the heterotypic LS174T/MRC-5 spheroids, the tumor cells (LoVo or LS174T) and the fibroblasts (MRC-5 or CCD-18Co) segregate in the two different compartments. The external peripheral layer of the tumor cells (CEA+) surrounds the central core of fibroblasts (FAP+), enriched in extracellular matrix mucopolysaccharidic components (ab-pas+). The tissue structure of the spheroid changes over time, as the external layer of tumor cells becomes thicker. The CD3, CD14, and CD45 staining confirm that the immune cells spontaneously infiltrate the spheroids. Homotypic spheroids with LS174T were not as compact as heterotypic spheroids and showed patchy CEA expression and similar basal lymphocyte infiltration compared to the heterotypic model.

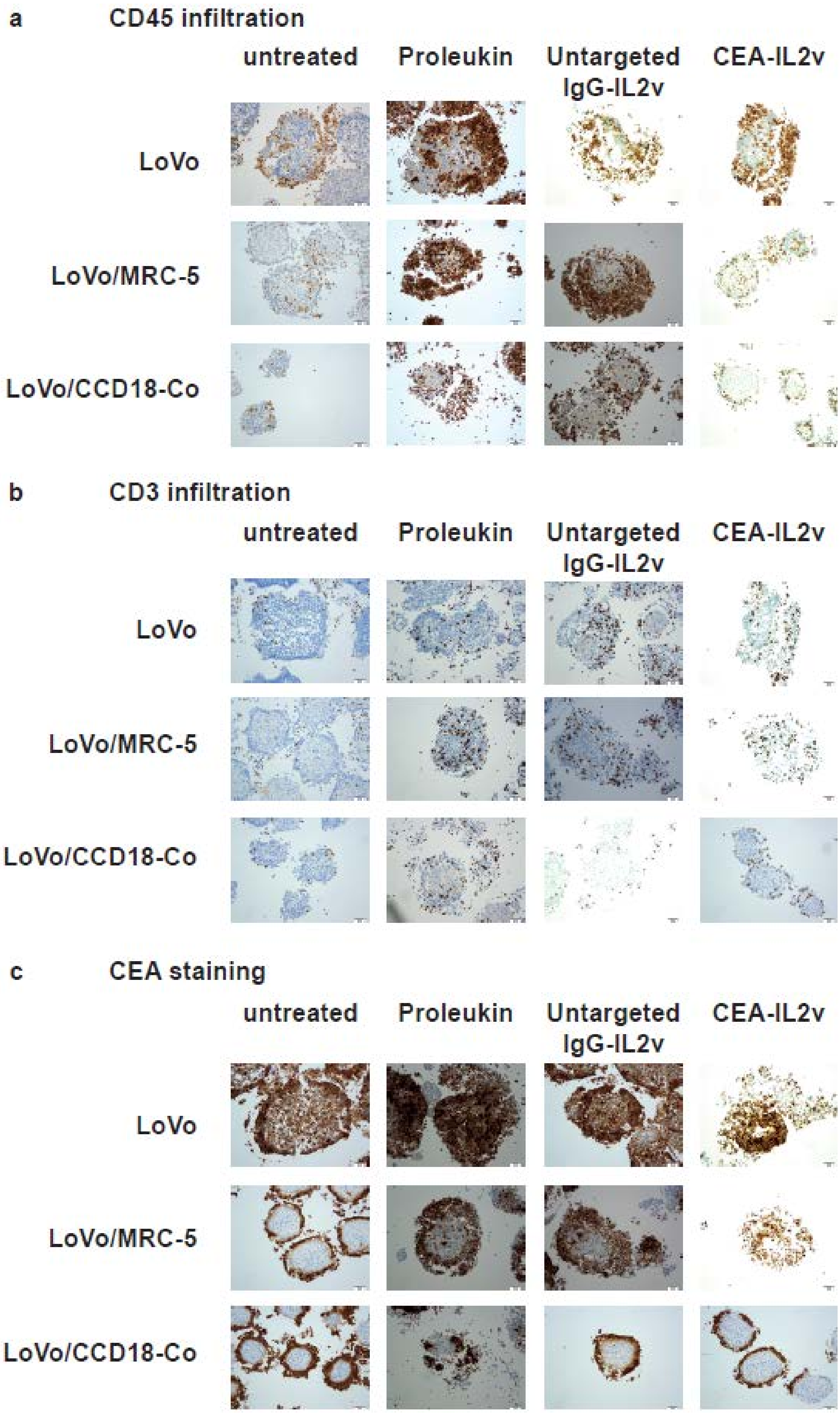

Supplementary Figure 2 Histology of homo- and heterotypic spheroids upon IL2 treatments. Stimulation of immune cell infiltration after 24-h incubation of the LoVo or heterotypic LoVo/MRC-5 or LoVo/CCD18-Co spheroids with 100 nM untargeted IgG-IL2v, Proleukin or CEA-targeted IL2v. Immunohistochemical CD45 (**a**), CD3 (**b**) and CEA (**c**) staining on 3-µm thick serial sections of the spheroids.

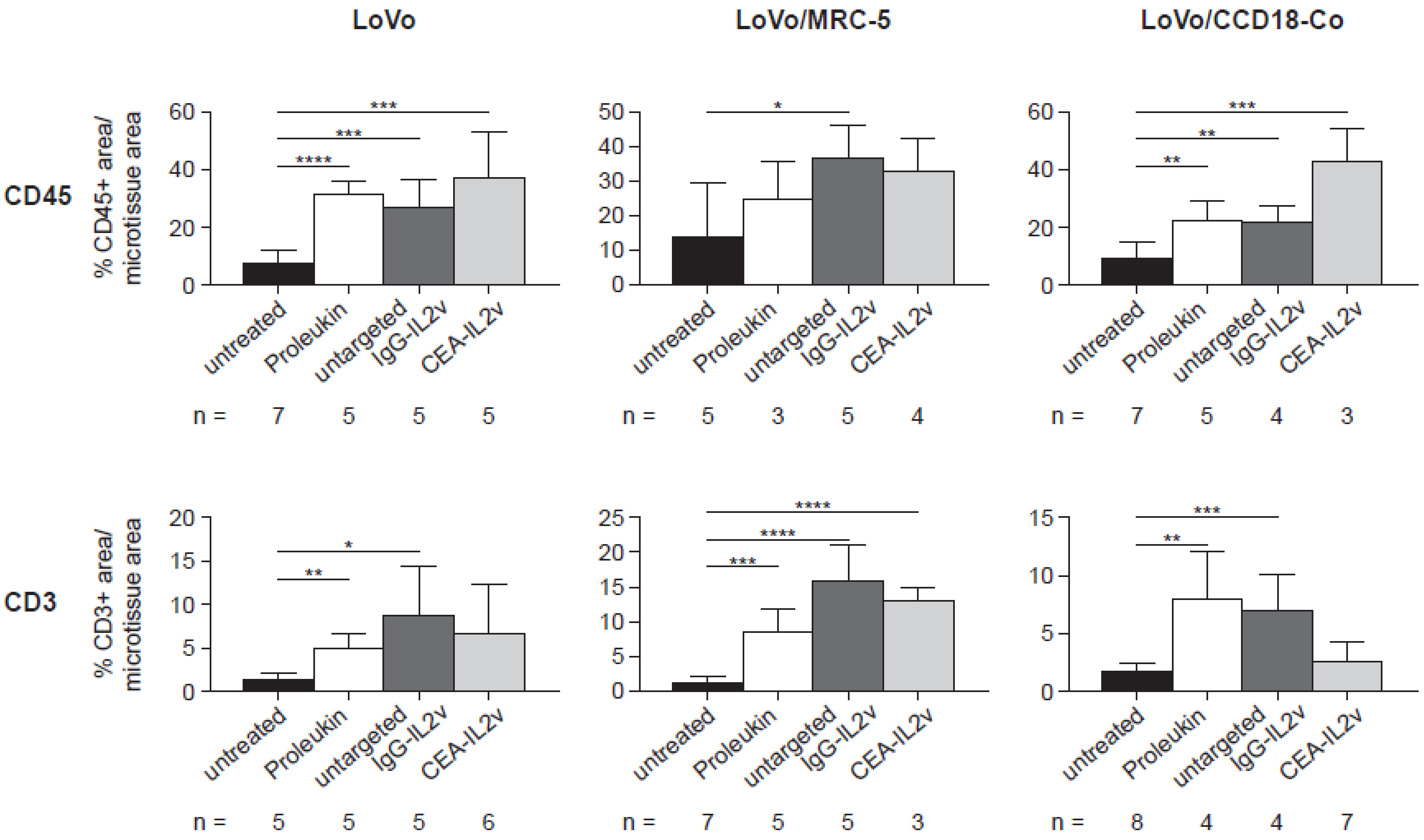

Supplementary Figure 3 Infiltration of lymphocytes upon treatment with different IL-2 molecules. Stimulation of immune cell infiltration after 24-h incubation of the LoVo or heterotypic LoVo/MRC-5 or LoVo/CCD18-Co spheroids with 100 nM untargeted IgG-IL2v, Proleukin or CEA-targeted IL-2v. The infiltration rates were calculated based on CD45+ leukocytes and CD3+ T cells staining normalized to the spheroid area (two-tailed unpaired *t*-test *p<0.05, **p<0.01, ***p<0.001, ****p≤0.0001).

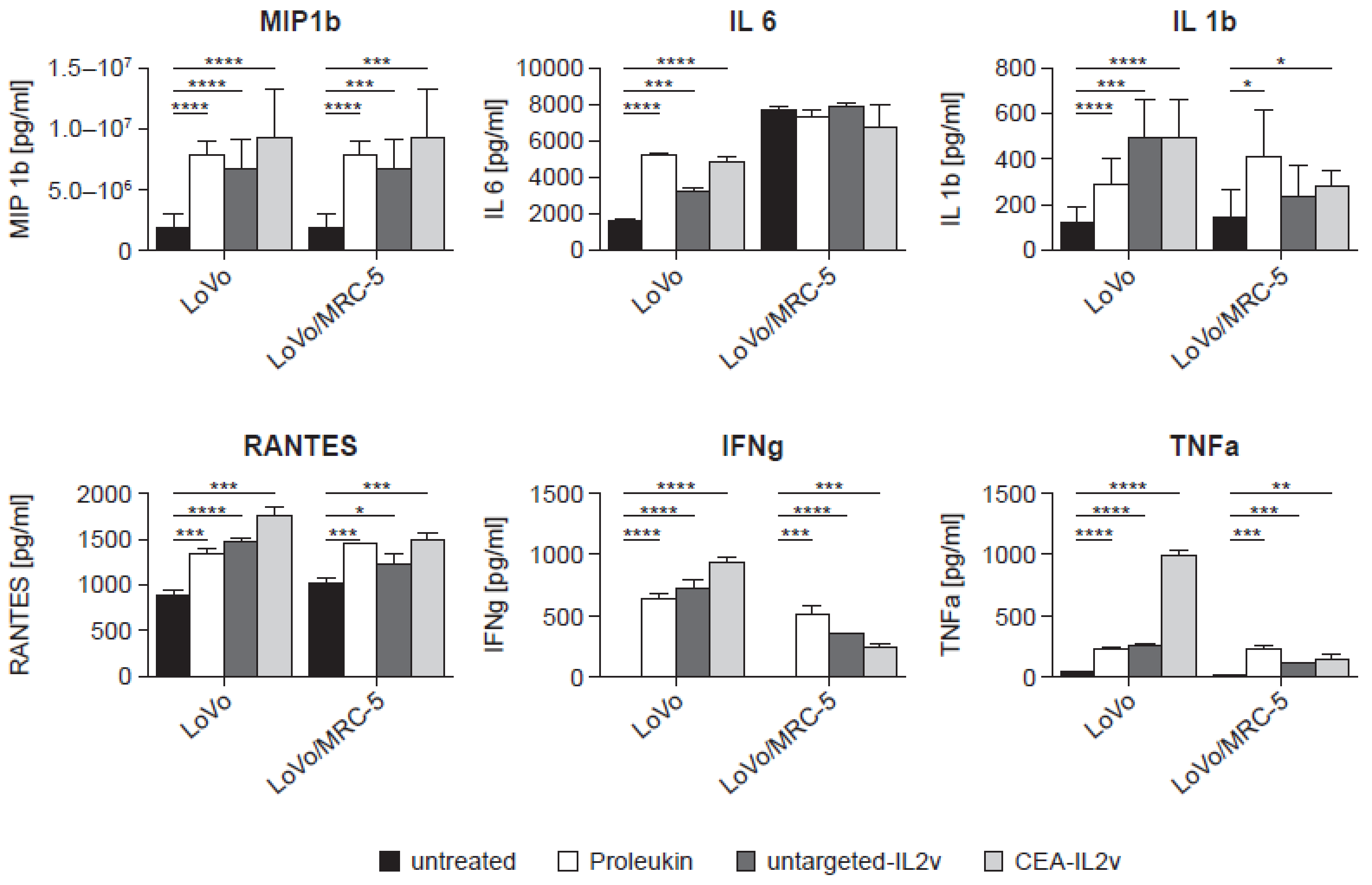

Supplementary Figure 4 Cytokine release and activation of infiltrated immune cells upon IL-2 treatments. Cytokine/chemokine secretion after 24-h treatment with 100 nM untargeted IgG-IL2v, Proleukin or CEA-targeted IL-2v of LoVo or heterotypic LoVo/MRC-5 spheroids (two-tailed unpaired *t*-test *p<0.05, **p<0.01, ***p<0.001, ****p≤0.0001; averages from triplicates of 2 donors).

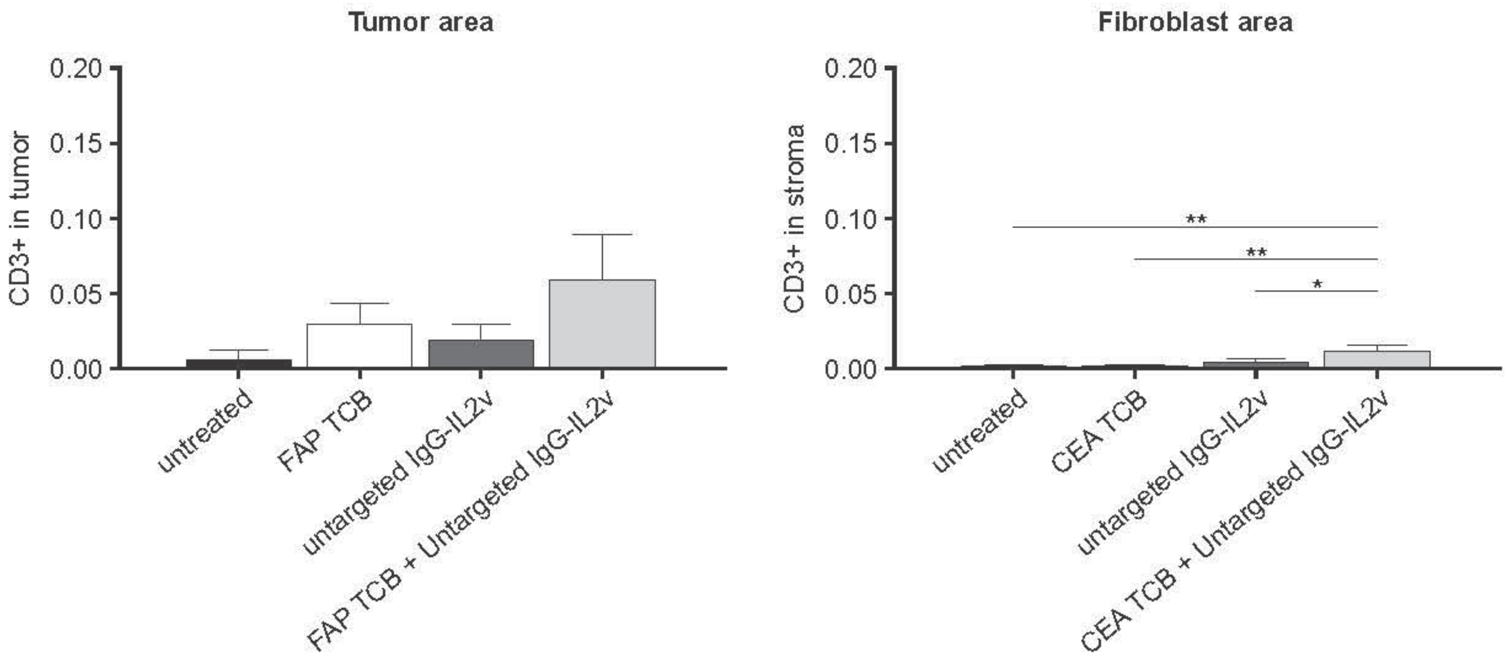

Supplementary Figure 5 Specificity of T cell cross-linking and retention in the heterotypic spheroid compartments. Quantification of T cells (CD3+ area, 3-µm thick serial sections) within the tumor and fibroblast areas of the heterotypic LS174T/MRC-5 spheroids at 24 h (two-tailed unpaired *t*-test *p<0.05, **p<0.01, ***p<0.001, ****p≤0.0001, N=3–4).
